# Perceived occupational health risks, noise and dust exposure levels among street sweepers in Mwanza City in Northern Tanzania

**DOI:** 10.1371/journal.pgph.0002951

**Published:** 2024-02-29

**Authors:** Elias C. Nyanza, Stella O. Jackson, Leah Magoha, Peter Chilipweli, Johannes Joshua, Monica T. Madullu

**Affiliations:** 1 Department of Environmental, Occupational and Research GIS, School of Public Health, Catholic University of Health and Allied Sciences, Mwanza, Tanzania; 2 Occupational Health and Safety Authority, Lake Zone Office, Mwanza, TANZANIA; 3 Department of Community Medicine, Catholic University of Health and Allied Sciences (CUHAS), Mwanza, TANZANIA; 4 Department of Microbiology and Immunology, Weill Bugando School of Medicine, Catholic University of Health and Allied Sciences, Mwanza, Tanzania; PLOS: Public Library of Science, UNITED STATES

## Abstract

Solid waste has been a major problem particularly in Sub-Saharan Africa countries as it has been increasing in several years. One of the methods employed in solid waste management is street sweeping which is done by street sweepers. Street sweepers are being predisposed to respiratory and non-respiratory infection like eye infection, skin infection and musculoskeletal disorders. This analytical cross-sectional study enrolled a total of 233 street sweepers to assess perceived occupational health risks, particulate matters (i.e., PM_2.5,_ PM_5_ and PM_10_ measured in (mg/m^3^)) dust and noise exposure levels in decibel units–dB(A), and utilization of protective personnel equipment among street sweepers in Mwanza city, Northern Tanzania. Modified poison regression was used to determine the association between exposures variables (i.e., sociodemographic, and socioeconomic factors) and outcomes ((i.e., noise dose level and particulate matters))). More than half (50.2%) of the participants were aged between 46–80 years. Their Median age was 46(IQR: 28–59) years. Large percent (63.1%) of the participants reported that their working environment have high health risks. The Median concentrations of the noise dose and particulates matters were 85.4 (IQR = 76.4–92.3) for noise dose, 13426 (IQR = 9637–17632) for PM_2.5_, 5522 (IQR = 2453–7679) for PM_**5**_, and 2310(IQR = 1263–3201). The Median concentrations of the noise dose and particulates matters were 85.4 (IQR = 76.4–92.3) for noise dose in decibel units dB(A); 13426 (IQR = 9637–17632) for PM_2.5_; 5522 (IQR = 2453–7679) for PM_5_; and 2310(IQR = 1263–3201) for PM_10_. Individual Street sweepers in Mwanza city are highly exposed to noise dose and fine particulate matters at levels above the reference values for human of 85dB(A) and 5mg/m^3^ respectively, making it a public health issue that requires holistic public health measures.

## 1. Introduction

Solid waste has been a problem in most African countries due to poor waste management. The mismanagement of solid waste in Africa has impacted human and environmental health. Data from 2012 showed that approximately 125 million tons per annum of municipal solid waste were generated in Africa in which 81 million tons were from Sub Saharan Countries [1]. This is expected to increase up to 244 million tons per year by 2025 [2]. One of the methods used in waste management is the—employment of street sweepers which helps to reduce the solid wastes in cities [3]. For instance, in Mwanza city council there is a constant population increase with a growth rate of 5.6% that has attracted production of large amounts of solid waste, leading to serious problems in the urban environment [4]. In response, the city has deployed street sweepers who play an important role in maintaining cleanness of the city, performing tasks such as picking up waste from its point of production, emptying refuse containers onto trucks, and delivering the waste to disposal and processing facilities [4].

Dust is considered as solid matter that is produced by any processes during mineral work, rock disintegration and any construction activities which is borne by air. Dust is referred to as small solid particles ranging from 1–75 mm [5]. In most of the developing countries, such as Tanzania, the use of machinery is minimal and/or inadequate, and most of the street cleaning is done by humans commonly called “*street sweepers*”. These groups of street sweepers are commonly exposed to occupational health hazards while attending their duties. For instance, a study in Nigeria reported street sweepers being exposed to occupation health risks like respiratory infection such as chronic obstructive pulmonary disease, coughing and non-respiratory infection like eye irritation [6]. Such occupational hazards have been reported to increase from time to time [7]. While implementing their activities, street sweepers have been reported to face several occupational health risks such as respiratory and non-respiratory infections, and noises. Such exposure to occupation health risks associated with street sweeping has been reported to increase the odds of health challenges [8].

The severity and onset of these health challenges may differ based on duration of exposure from dust, concentration and content of the dust, noises, vulnerability factors such as age and other co-morbidities [9]. A study conducted in India revealed that morbidities detected among street sweepers were anemia (20.5%), hypertension (9.5%), upper respiratory tract infections (7.3%) and chronic bronchitis (5.9%) [8].

Little attention has been given to explore the underlying reasons behind inefficient use of personal protective equipment (PPE), dust exposure levels and links to the occupational health risks which have been purported to be widely spread across the country especially around urban areas of Tanzania. In order to protect individuals involved in solid waste management (i.e., collection and disposal), the use of PPE is vital [10]. Whereas in any factory or workplace, workers who are employed in any process involving exposure to any injurious, offensive substance or environment, effective protective equipment shall be provided and maintained by employer for the use of the persons employed [11].

In Tanzania, the proper utilization of personnel protective equipment is clearly not understood, this may either be due to poor financial status or to the fact that they are not given to the street sweepers by the respective companies. Thus, having well informed data about the relationship of these dual phenomena could assist the government to enhance and advocate for the proper use of PPE among street sweepers which is a key strategy to reduce health risks [12]. Mwanza city is the second-largest urban settlement in Tanzania after the city of Dar es Salaam, with a population of over 900,000, and a major business center for regions around Lake Victoria and neighboring countries such as Kenya, Uganda, Burundi, and Rwanda. The city experiences large movements of people and car traffic throughout the year with both being associated with large production of waste such as fumes, aerosol, bio-aerosol, and dust which may increase risks of occupation hazards among street sweepers who are being exposed as they undertake their daily work duties. Also, in Mwanza city, less is known about the hazards and health effects encountered by street sweepers, and there are no general regulations in this field. In this context, the study has therefore been designed to assess the occupational health risks, dust exposure levels and the utilization of PPE among street sweepers in Mwanza city, Northern Tanzania.

## 2. Materials and methods

### 2.1. Study design and study settings

The study protocol for this analytical cross-sectional study was approved by the Joint Catholic University of Health and Allied Sciences and Bugando Medical Centre Research Ethics and Review Committee (CREC) (*certificate number CREC 2042/2021*). Permission to conduct the study was also obtained from the Regional and District health offices in Mwanza and Nyamagana Respectively. The study deployed both interviews and analytical components.

The study was carried out in Nyamagana municipal council in Mwanza city northwestern Tanzania from May to July 2022. Mwanza city is the second-largest urban settlement in Tanzania after the city of Dar es Salaam, with a population of over 900,000, and a major business center for regions around Lake Victoria and neighboring countries such as Kenya, Uganda, Burundi, and Rwanda. The city experiences large movements of people and car traffic throughout the year with both being associated with large production of waste such as fumes, aerosol, bio-aerosol, and dust which may increase risks of occupation hazards among street sweepers who are being exposed as they undertake their daily work duties.

### 2.2. Study population and participant recruitment

The study enrolled street sweepers from two cleaning companies namely (GIN Investment and Green Waste) provided they had been working on similar occupation for more than six months as are expected to have been more acquaintance with job experiences and challenges as suggested elsewhere [13]. Prior to participation in the study, participant information sheets explaining the purpose, benefit, risks, and rights for participation before being asked for consent were issued. A written informed consent was obtained from street sweepers who were willing to participate in the study. Street sweepers had the opportunity to ask questions before consenting to participation. All information from the participants was treated as confidential and used only for this study, and not otherwise. All interviews were carried out in privacy. Identification codes were used in the study instead of names and were not linked in any way with the information provided.

The minimum sample size for this study (n = 116) was determined based on a similar Indian study by Sabde et al., [14]. A total of 241 participants were enrolled in this study using stratified purposive sampling [15, 16]; 233 participants completed the face to face interview whereas 8 participants were not able to complete the face to face interviews and were excluded from the study.

### 2.3. Data collection

To ensure the quality of the results, different quality control measures were implemented. 1^st^—the study tools including questionnaires and checklist were pre-tested to ensure that the questions asked, and forms used were capturing the intended information and were understandable by study participants and staff involved. 2^nd^–to enhance accuracy and precision, all equipment used for noise levels and particulate matter measurement were calibrated and verified to account for possible field errors and to enhance accuracy of the analytical procedures.

#### 2.3.1. Socio-demographic and socio-economic information

Structured pretested questionnaires were used to collect social-demographic characteristics and reported perceived health challenges related to the study. The questionnaire on exposure hazards were adopted from previous studies in similar settings as reported elsewhere [13]. The socioeconomic status (SES) among street sweepers was estimated using traditional indicators of asset ownership like those used in Demographic and Health Surveys programs [17]; and presented as socioeconomic wealth quintile (SEWQ) [13]. Based on the Tanzanian context, asset ownership was based on pre-determined eight elements as reported elsewhere [13]; to determine the SEWQ which included; ownership of a house, materials used for housing construction, access to water for domestic use, access to electricity or solar energy at the household, number of meals normally eaten per day, permanent source of income, partner’s sources of income, and ownership of assets for transport [13].

#### 2.3.2. Data on occupational exposure

In occupational exposure as one of the outcome variables, we gathered data on noise level and particulate matters concentration, measured in decibels dB(A) and size of PM_2.5_ and PM_10_ measured in milligram per cubic meter (mg/m^3^) respectively. Exposure to noise levels and particulate matters was measured using a dosimeter and particulate counter (PCE-PCO 1), respectively. The Particulate counter (PCE-PCO 1) measures total dust, and other particulates of different sizes from 0.30 micrometers (μm) to 10μm and it gives direct result in display screen the concentration of fumes in mg/m^3^. Both instruments were calibrated according to the standardized manuals and procedures. For personal noise exposure, a dosimeter was calibrated using a standard tone of known pitch and intensity produced by the calibrator (i.e., 92db(A) at 1000Hz) as detailed in the handbook of Noise and vibration control [18]; whereas for particulate matters concentration, the Particulate counter (PCE-PCO 1) was calibrated and tested based on the particulate counter manual [19].

For personal noise exposure, a noise dosimeter was attached to the individual street sweeper’s belt or waistband and a small microphone connected to the dosimeter by a cord was fastened at the street sweepers clothing on the top of the shoulder at appointing midway between ear and shoulder. The dosimeter was typically set up to collect noise measurement results with different settings for noise exposure limits references to *OSHA Permissible Exposure Limit* (PEL) [20]. The instrument was set at the slow response, exchange rate 3 criterion level 90dB(A) and threshold level 80dB(A) [21]. Street sweepers stayed with the instrument throughout the work shift then after shift, the time-weighted average (TWA) in decibels was recorded directly from the instrument. This exercise was repeated for three consecutive days and an average was reported. Study participants were required to dress normally as per their routine protective measures.

In this study, only particulates size to be considered as 2.5, 5.0 and 10μm. The instrument was placed at a street where the respective street sweeper was working at 0.5–1.5 meters to measure particulate matters street sweepers are being exposed to. The instrument was calibrated at a flow rate of 2.83 liters per minute. Measurements were collected twice, before street sweeping activities and during street sweeping activities to find the average fumes concentration at each site and to minimize the effects of confounders.

### 2.4. Data management and data analysis

Data cleaning and analysis was performed using STATA version 15, Stata Corp LP [22]. Some of the variables like age, marital status, and socio-economic status were re-categorized based on previous literature for better interpretation and comparability between studies. The reproductive age group was defined at 18–45 years whereas older age at 46–80 years. Perceived occupational health risk was a composite variable based on six questions which are *i) Have you been cut and/or pricked by any sharp object found in the waste*? *ii) Do you cough more frequently at your work place*? *iii) Do you experience nose irritation at the working environment*? *iv) Do you experience eye discomfort during working*? *v) Do you have diseases that are perceived to be associated with your working environment vi) Do you experience shortness of breath during work*?. All six questions had the same weight. We scored “1” for “yes response” and “0” for “no response”. For analysis purpose, participants who scored above 75% on six questions were classified as experiencing high health risks in their working environment and participants who scored below 75% were classified as experiencing low health risk in their working environment.

In the descriptive statistics, continuous variables were summarized using median and interquartile range while categorical variables were summarized using frequency and percentages. The dependent variables (noise dose and particulate matters at size of PM_2.5,_ PM_5_ and PM_10_) were continuous. We checked for normality of outcome variables through density plots we found that they were both skewed. In the advanced analysis noise dose and fine particulate matters were categorized based on the reference values for human of 85dB(A) and 5 mg/m^3^ respectively. Our outcomes were very common (i.e., above 10% prevalent) thus Modified poison regression model was used to determine the association between exposures and outcomes of interest. Robust standard errors were used in the modified poison regression models to avoid overestimation of errors of the estimated prevalence ratios.

All independent variables with *p-*value <0.05 in the bivariate analysis were entered in a multivariable model to adjust for the confounding effect to figure out the real existing association between exposures and outcome of interest. The strength of association was expressed using prevalence ratio (PR) with their 95% confidence intervals. Independent variables with *p-*value <0.05 in multivariable analysis were considered as statistically significantly associated with the outcomes of interest. To build a model, stepwise regression was performed to assess the extent of association between variables.

## 3. Results

### 3.1. Background characteristics of the study participants

A total of 233 participants were enrolled in this study. The characteristics of the study participants are shown in Table 1. More than half (50.2%) of the participants were aged between 46–80; median 46(IQR: 28–59) years. Individual women sweepers were the dominating group as compared to male (56.6% vs 43.4%). Most of the street sweepers were neither married nor living with a partner (57.5%). Most of the participants had completed at least primary education (69.5%) and had 1–4 years at the workplace (79.8%). Majority of the street sweepers were of low socio-economic profile (51.9%) based on their SSE. Most of the street sweepers who participated in this study were not aware on the use of PPE (61.4%), even though they had reported to be using some of the PPE (93.1%). All the street sweepers participated in the present study, had no complete protection safety equipments as per standard guidelines, and none had ear plugs or any other equipment for noise protection.

**Table 1:** Sociodemographic characteristics of the study participants (N = 233).

Variables	n	%
*Age categories of the participants (years)*		
18–45	116	49.8
46–80	117	50.2
Median age (IQR)	46(28–59)	
Min age: 18years; Max age: 80years		
*Sex*		
Male	101	43.4
Female	132	56.6
*Marital status*		
Married	99	42.5
Single/Separated/Widow/Widower	134	57.5
*Education level*		
Not gone to school	71	30.5
At least primary education	162	69.5
*Social economic wealth Quintile (SEWQ)*		
High	53	22.8
Moderate	59	25.3
Low	121	51.9
*Duration at work(years)*		
Less than 1 year	30	12.9
1–4 years	186	79.8
5–8 years	17	7.3
*PPE awareness*		
Awareness (≥50%)	90	38.6
Unawareness (<50%)	143	61.4
*Frequency of PPE use*		
All the time	217	93.1
Not all the time	16	6.9
*Gained training on health and safety at work*		
Yes	38	16.3
No	195	83.7
*Employer*		
Green Waste	124	53.2
GIN	109	46.8

Note: None of the street sweepers had complete safety equipments, and none had ear plugs for ear protection.

### 3.2. Reported perceived persistent occupational health challenges among participants

The reported perceived occupational health risks among participants are detailed in Table 2. Most of the study participants reported to have experienced road traffic accidents at some point while on duty (79.4%). Few participants reported to have experienced cut or pricked by sharp objects (22.3%). eye problems (14.6%), nose irritation (7.3%), difficulty during breathing (3.9%), and coughing (3.4%).

**Table 2:** Reported perceived persistent occupational health challenges among participants (N = 233).

Variables	n	%
*Type of accident that encountered frequently at work*		
Road Traffic Accidents (RTA)	185	79.4
Fall	13	5.6
RTA/Fall/needle/glasses	12	5.2
Can’t Recall	23	9.8
*Having diseases associated with your work*		
Yes	103	44.2
No	130	55.8
*Trouble breathing*		
Yes	9	3.9
No	224	96.1
*Eye problems*		
Yes	34	14.6
No	199	85.4
*Nose irritation*		
Yes	17	7.3
No	216	92.7
*Coughing*		
Yes	8	3.4
No	225	96.6
*Cut/pricked by sharp objects found in wastes*		
Yes	52	22.3
No	181	77.7

### 3.3. Occupational exposure levels to noise dose in dB(A) and concentration of particulates matters size of PM_2.5_, PM_5_ and PM_10_ in (mg/m^3^) among study participants stratified by age

The median concentrations of the noise dose and particulates matters were 85.4 (IQR = 76.4–92.3) dB(A) for noise dose, 13426 (IQR = 9637–17632) for PM_2.5_, 5522 (IQR = 2453–7679) for PM_5_, and 2310 (IQR = 1263–3201) for PM_10_. Most of the participants were highly exposed to noise dose (≥85dB: n = 117, 50.6%), PM_2.5_ (5: n = 215, 92.3%), PM_5_ (5: n = 148, 63.5%) and PM_10_ (5: n = 215, 92.3%) (Table 3). After stratification based on age status, majority of the reproductive age participants were found to be highly exposed with PM_2.5_ (93.1%), PM_5_ (74.1%) and PM_10_ (93.1%). Moreover, for noise dose more than half of the participants with old age (54.8%) were highly exposed (Table 3).

**Table 3:** Occupational exposure levels to noise dose in dB(A) and particulates matter levels at size of PM_2.5_, PM_5_ and PM_10_ stratified by age.

Variables	Overall		Age status				P10	P25	Median(95%CI)	P75	P95	Max
			*Reproductive age*		*Old age*							
	n	%	n	%	n	%						
**Noise Dose dB(A)**							71.6	76.4	85.4(76.1–86.7)	92.3	98.3	99.4
Less exposed (≤85)	114	49.4	62	53.5	52	45.2						
Highly exposed (>85)	117	50.6	54	46.5	63	54.8						
**PM**_**2.5**_ **(mg/m** ^**3**^ **)**							5326	9637	13426(1211–13734)	17632	21636	21736
Less exposed (≤5)	18	7.7	8	6.9	10	8.5						
Highly exposed (>5)	215	92.3	108	93.1	107	91.5						
**PM**_**5**_ **(mg/m**^**3**^**)**							703	2453	5522(5412–5634)	7679	9326	9426
Less exposed (≤5)	85	36.5	30	25.9	55	47.0						
Highly exposed (>55)	148	63.5	86	74.1	62	53.0						
**PM**_**10**_ **(mg/m**^**3**^**)**							276	1263	2310(2134–2536)	3201	3807	6411
Less exposed (≤5)	18	7.7	8	6.9	10	8.6						
Highly exposed (>5)	215	92.3	108	93.1	107	91.4						

Dichotomization was done using the reference values of 85 for noise and 5 for particulate matter measured in decibels dB(A) and milligram per cubic meter (mg/m^3^) respectively.

### 3.4. Adjusted association between exposure variables and noise dose noise dose in dB(A) and PM_2.5_

In the adjusted analysis, duration at the working place, difficulty in breathing as well as nose irritation were significantly associated with prevalence of noise dose (Table 4). Participants who had worked for 1–4 years had 15% higher prevalence of noise dose (aPR = 1.15, 95%CI = 1.03–2.08) as compared to those who had worked for less than one year; participants who reported to have trouble during breathing had 47% higher prevalence of noise dose (aPR = 1.47, 95%CI = 1.21–1.79) as compared to their counterparts who had no trouble during breathing and participants with nose irritation had 9% higher prevalence of noise dose (aPR = 1.09, 95%CI = 1.01–1.91) as compared to their counterparts who had no nose irritation (Table 4).

**Table 4:** Adjusted association between exposure variables and noise dose in dB(A) and particulates matters at size of PM_2.5_.

Variables	Noise dose(dB) PM_2.5_ (mg/m^3^)					
	aPR	95%CI	P-value	aPR	95%CI	P-value
**Age categories of the participants (years)**						
18–45	1.18	(0.21,1.77)	0.19	1.15	(1.10,1.83)	0.17
46–80		**Ref**			**Ref**	
**Sex**						
Male		**Ref**			**Ref**	
Female	1.05	(0.93,1.17)	0.44	1.03	(1.01,1.96)	0.01
**Marital status**						
Married		**Ref**			**Ref**	
Single/Separated/Widow/Widower	0.99	(0.89,1.09)	0.87	1.01	(0.85,1.19)	0.88
**Education level**						
Not gone to school	0.94	(0.84,1.04)	0.20	1.07	(0.93,1.23)	0.35
At least primary education		**Ref**			**Ref**	
**Social economic wealth Quintile**						
High	1.09	(0.01,1.56)	0.32	1.34	(0.03, 1.89)	0.20
Moderate		**Ref**			**Ref**	
Low	0.83	(0.71,1.09)	0.43	1.67	(1.02,1.78)	0.03
**Duration at work(years)**						
Less than 1 year		**Ref**			**Ref**	
1–4 years	1.15	(1.03,2.08)	0.03	0.87	(0.73,1.05)	0.15
5–8 years	0.89	(0.73,1.09)	0.28	1.49	(1.34,1.69)	<0.001
**PPE awareness**						
Awareness (≥50%)	0.90	(0.82,0.99)	0.02	1.04	(0.92,1.18)	0.52
Unawareness (<50%)		**Ref**			**Ref**	
**Utilized PPE**						
Yes	0.59	(0.40,0.96)	0.04	0.73	(0.15,1.78)	0.08
No		**Ref**			**Ref**	
**Frequency of PPE use**						
All the time	1.08	(0.91,1.28)	0.39	1.09	(0.86,1.38)	0.49
Not all the time		**Ref**			**Ref**	
**Gained training on health and safety at work**						
Yes	0.97	(0.86,1.11)	0.74	1.03	(0.89,1.19)	0.67
No		**Ref**			**Ref**	
**Type of accident that encountered frequently at work**						
RTA		**Ref**			**Ref**	
Fall	0.90	(0.76, 1.07)	0.23	0.85	(0.68,1.06)	0.15
RTA/Fall/needle/glasses	0.69	(0.59,0.81)	<0.001	0.74	(0.58,0.93)	0.01
**Having diseases associated with your work**						
Yes	1.13	(0.05,1.73)	0.50	1.21	(045,1.78)	0.23
No		**Ref**			**Ref**	
**Trouble breathing**						
Yes	1.47	(1.21, 1.79)	<0.001	1.41	(1.07,1.85)	0.02
No		**Ref**			**Ref**	
**Eye problems**						
Yes	1.02	(0.91,1.14)	0.76	1.79	(1.05,2.98)	0.03
No		**Ref**			**Ref**	
**Nose irritation**						
Yes	1.09	(1.01,1.91)	0.001	0.84	(0.63,1.12)	0.24
No		**Ref**			**Ref**	
**Coughing**						
Yes	1.14	(1.05,1.71)	0.05	1.22	(0.89,1.67)	0.21
No		**Ref**			**Ref**	
**Cut/pricked by sharp objects found in waste**						
Yes	1.01	(0.91,1.13)	0.84	1.14	(0.99,1.29)	0.06
No		**Ref**			**Ref**	
**Perception on health risks**						
High	1.52	(0.17,1.64)	0.25	1.48	(0.18,1.73)	0.16
Low		**Ref**			**Ref**	

Also, Sex, duration at the workplace, SSE, trouble during breathing and eye problems were significantly associated with prevalence of PM_2.5_. Female participants had 3% higher prevalence of PM_2.5_ (aPR = 1.03, 95%CI = 1.01–1.96) as compared to male participants; Participants who had worked for 5–8 years had 49% higher prevalence of PM_2.5_ (aPR = 1.49, 95% CI = 1.34–1.69) as compared to those who had worked for less than one year. Participants with low SSE had 67% higher prevalence of PM_2.5_ (aPR = 1.67, 95%CI = 1.02–1.78) as compared to participants with moderate SES. Moreover, participants who reported to have trouble during breathing had 41% higher prevalence of PM_2.5_ (aPR = 1.41, 95%CI = 1.07–1.85) as compared to their counterparts who reported to have no trouble during breathing process and participants who had eye problems had 79% higher prevalence of PM_2.5_ (aPR = 1.79, 95%CI = 1.05–2.98) as compared to their counterparts (Table 4).

### 3.5. Adjusted association between exposure variables and particulates matters at the size of PM_5_ and PM_10_

In the adjusted analysis, Age, duration at work, frequency of PPE use, trouble during breathing and eye problems were significantly associated with prevalence of particulate matters at PM_5_ (Table 5). Participants of reproductive age had 28% higher prevalence of PM_10_ (aPR = 1.28, 95% CI = 1.16–1.90) as compared to participants with older age; Participants who had worked for 5–8 years at the workplace had 29% higher prevalence of PM_5_ (aPR = 1.29, 95% CI = 1.17–2.94) as compared to their reference group worked for less than one year. Interesting that participants who use PPE all the time during working hours had 31% higher prevalence of PM_5_ (aPR = 1.31, 95% CI = 1.09–1.57) as compared to their control who do not use PPE all the time. Participants who had trouble during breathing had 47% higher prevalence of PM_5_ (aPR = 1.47, 95% CI = 1.20–1.78) as compared to their reference group, Moreover, participants with eye problems had 56% higher prevalence of PM_5_ (aPR = 1.56, 95% CI = 1.07–1.98) as compared to their reference group with no eye problems (Table 5).

**Table 5:** Adjusted association between exposure variables and particulates matters at the size of PM_5_ and PM_10_.

Variables	PM_5_ (mg/m^3^) PM_10_ (mg/m^3^)					
	aPR	95%CI	P-value	aPR	95%CI	P-value
**Age categories of the participants(years)**						
18–45	1.28	(1.16,1.90)	<0.001	1.31	(1.12,1.67)	0.04
46–80		**Ref**			**Ref**	
**Sex**						
Male		**Ref**			**Ref**	
Female	0.92	(0.84,1.02)	0.12	1.16	(1.09,1.93)	<0.001
**Marital status**						
Married		**Ref**			**Ref**	
Single/Separated/Widow/Widower	0.97	(0.89,1.06)	0.55	1.01	(0.93,1.10)	0.82
**Education level**						
Not gone to school	0.97	(0.88,1.06)	0.50	0.96	(0.88,1.04)	0.31
At least primary education		**Ref**			**Ref**	
**Social economic wealth Quintile**						
High	1.45	(0.23,1.76)	0.34	1.60	(0.04, 1.89)	0.3
Moderate		**Ref**			**Ref**	
Low	0.56	(0.41,1.56)	0.21	1.42	(1.04,1.59)	0.04
**Duration at work(years)**						
Less than 1 year		**Ref**			**Ref**	
1–4 years	0.97	(0.87,1.08)	0.57	0.97	(0.88,1.08)	0.59
5–8 years	1.29	(1.17,2.94)	0.01	1.53	(1.07,2.95)	0.02
**PPE awareness**						
Awareness (≥50%)	1.05	(0.97,1.13)	0.26	0.94	(0.87,1.02)	0.13
Unawareness (<50%)		**Ref**			**Ref**	
**Utilized PPE**						
Yes	0.65	(0.41,0.91)	0.03	0.49	(0.18,1.76)	0.07
No		**Ref**			**Ref**	
**Frequency of PPE use**						
All the time	1.31	(1.09,1.57)	0.004	1.07	(0.98,1.16)	0.12
Not all the time		**Ref**			**Ref**	
**Training on health and safety at work**						
Yes	0.96	(0.88,1.05)	0.40	1.01	(0.93,1.08)	0.88
No		**Ref**			**Ref**	
**Type of accident that encountered frequently at work**						
RTA		**Ref**			**Ref**	
Fall	0.92	(0.76,1.10)	0.35	1.12	(0.98,1.28)	0.11
RTA/Fall/needle/glasses	0.87	(0.69,1.09)	0.24	0.97	(0.85,1.10)	0.63
**Having diseases associated with your work**						
Yes	0.77	(0.54,1.91)	0.3	0.69	(0.45,1.23)	0.34
No		**Ref**			**Ref**	
**Trouble breathing**						
Yes	1.47	(1.20,1.78)	<0.001	1.24	(0.95,1.62)	0.11
No		**Ref**			**Ref**	
**Eye problems**						
Yes	1.56	(1.07,1.98)	0.01	0.87	(0.75,1.00)	0.06
No		**Ref**			**Ref**	
**Nose irritation**						
Yes	0.76	(0.65,1.89)	0.2	0.84	(0.64,1.11)	0.22
No		**Ref**			**Ref**	
**Coughing**						
Yes	0.74	(0.58,1.93)	0.1	0.79	(0.56,1.09)	0.16
No		**Ref**			**Ref**	
**Cut/pricked by sharp objects found in waste**						
Yes	0.92	(0.83,1.01)	0.08	1.05	(0.98,1.13)	0.15
No		**Ref**			**Ref**	
**Perception on health risks**						
High	1.64	(1.17,1.78)	0.02	1.45	(1.14,1.94)	0.01
Low		**Ref**			**Ref**	

After adjusting for other variables, Age, Sex, SES, and duration at the workplace were significantly associated with prevalence of particulate matters at PM_10_. Participants of reproductive age had 31% higher prevalence of PM_10_ (aPR = 1.31, 95% CI = 1.12–1.67) as compared to participants with older age; Female participants had 16% higher prevalence of PM_10_ (aPR = 1.16, 95%CI = 1.09–1.93) as compared to male participants. Participants with low SES had 42% higher prevalence of PM_10_ (aPR = 1.42, 95%CI = 1.04–1.59) as compared to participants with moderate SES. However, participants who had worked for 5–8 years at the workplace had 53% higher prevalence of PM_10_ (aPR = 1.53, 95%CI = 1.07–2.95) as compared to those who had worked for less than one year (Table 5).

## 4. Discussion

Street sweeping is one of the neglected occupations in Tanzania among other LMICs. Such vulnerable and marginalized group are potentially exposed to a numbers of health hazards. The primary work of street sweeping, and waste collecting are conducted while involving underprivileged individuals. Thus, the aim of this study was to assess perceived occupational health risks, dust and noise exposure levels and utilization of protective personnel equipment among street sweepers in Mwanza city.

The majority of street sweepers who participated in this study were female (56.6 vs 43.4%), this is consistent with other studies conducted in India and Western Nigeria where 67.1% and 100% of the street sweepers were female respectively [6]. Indicating this industry is dominated by women as compared to men. Also, most of the participants only had primary education (69.5%) and did not develop their education further. The high proportion of participants (61.4%) being unaware on proper PPE use and the findings that 83.7% of the study participants had no proper training on safety measures during work, calls for immediate attention. However, designing and provision of education kits and preventive measures on occupational protective measures should also consider the level of education as the majority are of low education status. Not having adequate education could limit an individual’s access to and comprehension of information on ways to avoid exposure to their respective occupation hazards [23].

Our findings indicate that a large number of the street sweepers (51.9%) involved in this study were of low socio-economic status based on their SEWQ. Lower socio-economic status has been associated with increased exposure to environmental toxicants including dust [17, 24, 25]. In the present study, exposure to PM2.5 particulate matters was more prevalent by 2% among individuals ‘street sweepers with low SEWQ. Studies have suggested that SES may limit lifestyle choices including day-to-day activities as well as dietary options [17, 24]; which could be potentiating the effect of exposure to particulate matters even at low doses.

This study observed that a high proportion of the study participants has potential exposure to high concentrations of particulate matters (92.3% at PM_2.5_, 63.5% at PM_5_, and 92.3% at PM_10_). Studies in different parts of the world have established the association between the exposure to particulate dust matters especially PM_2.5_ with a risk of developing coronary diseases, lung diseases and poor lung function [26, 27]. Without appropriate dust protective equipments, particulate matters at PM_2.5_ size can penetrate deep into the lungs, irritate, and corrode the alveolar walls and consequently impair the lung function [28]. Most of the street sweepers were at the most susceptible age to infections–immunoscenescence–diminished immune response against infection, increased levels of pro-inflammatory mediators and increased risk of autoimmunity which are observed commonly in elderly population [29]. With increased exposure to fine particulate matters such as PM2.5, such individuals could have a potentiated effect not only to infection, but also to non-infectious diseases [29]. In Tanzania, the retirement age is up between 60 and 65 years, it is unfortunately most of the street sweepers are engaged in such activities at post-retirement age (i.e., above 65 years), making them more prone to diseases. It is important for authorities to enforce employment policies and regulations to this particular industry regarding employment age limit.

For reported perceived occupational risk among street sweepers, the majority of the individual street sweepers reported to have experienced road traffic accidents while attending their duties. In addition, a substantial proportion of the study participants reported to have experienced diseases associated with their work including musculoskeletal pain especially back pain and leg pain due constantly standing and bending, as well as eyes and nose irritation. A considerable proportion of the street sweepers reported to have been cut or pricked by objects during waste handling, had eye problems, being irritated by noise, and had difficulty in breathing at 22.3%, 14.6%, 7.3% and 3.9% respectively. A recent systematic review reported that musculoskeletal problems, shoulder pain, respiratory disorder, cuts slips, and RTA are the most common work-related complaints among street sweepers [6]. Our findings also indicated PM2.5 particulate matters exposure being 41% and 79% more prevalent among those with reported difficulty in breathing and eye problems, respectively. Our findings are in line with those published elsewhere in India where a high proportion of work related health issues such as allergies, work related injuries and respiratory disorders reported to be ranging from 12.8% to 48.9% with poor PPE usage, poor knowledge and attitude on the use of PPE being among the associated factors [23].

Exposure to noise levels above 85dB(A) during their working days among street sweepers in the present study provide an alert to a silent yet significant occupation health problem among those involved. According to OSHA, exposure to noise should not exceed 100dB(A), and city traffic noise should not exceed 85dB(A) [20, 30]. Street sweepers in the present study are exposed to noise levels above 85dB(A) for more than 8hours a day. This among other reasons could be potentiating the risk for hearing loss [30], There is sufficient scientific evidence that noise exposure can induce hearing impairment, hypertension and ischemic heart disease, annoyance, sleep disturbance, and decreased school performance, therefore this may be of serious consequence if not addressed [30–32]. In the present study, exposure to noise levels above 85dB(A) was more prevalent among street sweepers who had reported difficulty in breathing, coughing, and nose irritation. Individual street sweepers utilizing at least partial PPE had a 41% protective effect against noise exposure. Our findings indicate that street sweepers may be at a higher risk for occupational hazard exposure such as accidents, noise, and diseases due to lack of complete PPE utilization, lack of proper PPE utilization and lack of knowledge and awareness on the proper PPE usage. Findings elsewhere have reported moderate availability of mouth/nose mask while respirators were completely unavailable [9]. It was unfortunate that, none of the street sweepers had complete safety equipment, and none had ear plugs or any other measures for ear protection against noise hazards. A recent systematic review remarked that, street sweepers in developing countries, are highly exposed to dust as there are no suitable protective measures available [6]. According to Occupational Health and Safety Authority (OSHA), employers are required to implement a hearing conversation program when the noise exposure is at or above 85 decibels dB(A) averaged over 8 working hours, such programs are lacking among street sweepers. Also wearing depends on attitude, knowledge and awareness on the risks and health adverse effects.

These findings are eye-opening and calls for more efforts in the training and health promotion. Findings from our study are in agreement with those in Dhaka city, Bangladesh where lack of protective equipment and limited usage of PPE among street sweepers was associated with poor health status, low work security and poor work performance [33].

Based on the current findings, there is a need for a ffollow-up longitudinal study to establish the association between PPE usage, particulate matters, and noise exposure levels with the potentiated health outcome among street sweepers. The high levels of particulate matters and noise dose that can contributed to acquiring respiratory and hearing problem respectively, authorities should add a provision of tasking a mandatory provision of adequate protective equipments to hired street sweepers among the awarded companies. It is important to conduct on-the-job training from time to time to raise awareness and understanding on PPE use. Since some of the street sweepers reported some cuts associated with solid waste collection, and we did not find any record for preventive vaccine, we recommend a “*Tetanus Toxoid injection - a toxoid vaccine used to prevent tetanus*” to all street sweepers prior to job engagement as are prone to cuts from waste collection.

This study has several limitations. First, we did not perform lung tests since this study was conducted during COVID-19 pandemic, so sharing of lung test devices would create some potential infection transmission. Secondly, Traffic Road Accidents and injuries were based on the reported findings from the street sweepers and were not verified against reported police cases. There is a possibility that we have underreported such cases. A comparison with police reports should be made in the future for monitoring purposes. Some of the street sweepers were afraid to participate and share their health issues as well as challenges due to the fear of losing their jobs. The content of dust is of paramount importance, and one should not under emphasize it. This study did not analyze the content of the particulate matter that street sweepers are being exposed to, which could have provided toxicological information beyond respiratory risks. A follow up study is therefore needed to examine the toxicological content of the particulate matters. Our findings coincide with previous published evidence elsewhere; however, generalizability should be restricted to the study settings and participants characteristics.

To the best of our understanding, this is the very first study to assess particulate matters levels and noise dose exposure among street sweepers in Tanzania. Examining both particulate matters levels and noise dose exposure provided information on dual exposure to two hazards among street sweepers in northern part of Tanzania.

## 5. Conclusion

Individual street sweepers in Mwanza city are highly exposed to noise dose and fine particulate matters at levels above the reference values for human of 85dB(A) and 5 mg/m^3^ respectively, making it a public health issue that requires urgent measures. Perceived occupational health challenges are common among street sweepers of which most are of older ages. Awareness on the use of PPE is inadequate among this group. Holistic public health measures are needed to address the challenge among the vulnerable population.

## Supporting information

S1 Data Data set. (CSV)

S1 File STROBE checklist. (DOCX)
